# Patterned frequency-modulated oral stimulation in preterm infants: A multicenter randomized controlled trial

**DOI:** 10.1371/journal.pone.0212675

**Published:** 2019-02-28

**Authors:** Dongli Song, Priya Jegatheesan, Suhas Nafday, Kaashif A. Ahmad, Jonathan Nedrelow, Mary Wearden, Sheri Nemerofsky, Sunshine Pooley, Diane Thompson, Daniel Vail, Tania Cornejo, Zahava Cohen, Balaji Govindaswami

**Affiliations:** 1 Pediatrics—Neonatology, Santa Clara Valley Medical Center, San Jose, CA, United States of America; 2 Stanford University School of Medicine, Palo Alto, CA, United States of America; 3 Pediatrics—Neonatology, Children's Hospital at Montefiore-Weiler Division, Albert Einstein College of Medicine, Bronx, NY, United States of America; 4 Pediatrix Medical Group, North Central Baptist Hospital, San Antonio, TX, United States of America; 5 Pediatrics–Neonatology, Baylor College of Medicine, San Antonio, TX, United States of America; 6 Pediatrics–Neonatology, Cook Children's Medical Center, Fort Worth, TX, United States of America; 7 Pediatrics–Neonatology, Children's Hospital at Montefiore-Wakefield Division, Albert Einstein College of Medicine, Bronx, NY, United States of America; 8 aVenture Consulting, LLC, Leawood, KS, United States of America; 9 Neonatology, Montefiore Medical Center-Weiler, Bronx, New York, United States of America; 10 Neonatology, Montefiore Medical Center-Wakefield, Bronx, New York, United States of America; KU Leuven, BELGIUM

## Abstract

**Objective:**

To evaluate the effect of patterned, frequency-modulated oro-somatosensory stimulation on time to full oral feeds in preterm infants born 26–30 weeks gestation.

**Study design:**

This is a multicenter randomized controlled trial. The experimental group (n = 109) received patterned, frequency-modulated oral stimulation via the NTrainer system through a pulsatile pacifier and the control group (n = 101) received a non-pulsatile pacifier. Intent-to-treat analysis (n = 210) was performed to compare the experimental and control groups and the outcomes were analyzed using generalized estimating equations. Time-to-event analyses for time to reach full oral feeds and length of hospital stay were conducted using Cox proportional hazards models.

**Results:**

The experimental group had reduction in time to full oral feeds compared to the control group (-4.1 days, HR 1.37 (1.03, 1.82) p = 0.03). In the 29–30 weeks subgroup, infants in the experimental group had a significant reduction in time to discharge (-10 days, HR 1.87 (1.23, 2.84) p < 0.01). This difference was not observed in the 26–28 weeks subgroup. There was no difference in growth, mortality or morbidities between the two groups.

**Conclusions:**

Patterned, frequency-modulated oro-somatosensory stimulation improves feeding development in premature infants and reduces their length of hospitalization.

**Trial registration:**

ClinicalTrials.gov NCT01158391

## Introduction

Feeding development undergoes a series of maturational processes throughout gestation, from non-coordinated sucking and swallowing movements to fully coordinated suck-swallow-breathe that usually occurs after 34 weeks gestation [1–4]. Unlike term or late preterm infants, very premature infants require parenteral nutrition and gavage feeding prior to reaching maturity for safe and independent oral feeding [5, 6]. During the transition from gavage to full oral feeds (FOF), which may take weeks to months, these infants have limited opportunities for suck and swallow practice and are subjected to frequent non-physiological orofacial stimuli while receiving necessary medical interventions such as endotracheal intubation, continuous positive airway pressure, oral and nasopharyngeal suctioning, and adhesive tapes limiting facial movements. These adverse experiences during a critical period for oral sensory-motor-brain development often result in delayed or abnormal feeding behaviors [7, 8]. Feeding difficulty is a significant contributor to prolonged length of stay (LOS) in the neonatal intensive care unit (NICU) [9]. The negative impact of feeding difficulties may persist into childhood, leading to growth failure and poor neurodevelopmental outcomes [10–13], particularly delayed language development [14, 15].

Oral feeding is a complex process which involves suck-swallow-breathe coordination, cardiorespiratory stability, behavioral state organization and neuromuscular support. The progression of feeding maturation follows the Synactive Theory in which the developing fetus/infant integrates multisensory inputs from the environment with internal physiological status and functional demands [16, 17]. Several entrainment strategies have been used to provide developmentally appropriate oral, auditory, olfactory, tactile/kinesthetic and vestibular sensory inputs to facilitate feeding development in the preterm infant [4, 18–25]. Among these interventions, oral stimulation is the method most studied and may include using a pacifier offered by caregivers [4, 18], or oral motor interventions performed by therapists [19, 26]. Oral stimulation has been shown to improve sucking skills and feeding performance [27–31], shorten the time to achieve FOF and reduce LOS in the NICU [4,18, 19, 26). However, some of the beneficial effects were not observed consistently [32–35]. To enhance the efficacy and consistency of oral stimulation via pacifier, Barlow et al. developed a bedside device, the NTrainer system, which delivers consistent patterned and frequency-modulated oro-somatosensory stimulation (PFOS) through a pneumatically pulsed pacifier interface [36]. PFOS has been shown to be more effective than a regular non-pulsatile pacifier in improving preterm infants’ sucking and feeding performance [36, 37]. Thus, PFOS represents a potentially effective therapy for helping preterm infants achieve independent oral feeds. The objective of this multicenter randomized controlled trial was to test the hypothesis that preterm infants who received the PFOS training would transition to FOF earlier than those who received oral suck training with non-pulsatile pacifiers.

## Methods

This multicenter randomized controlled trial was conducted from 2011 to 2015 in five NICUs (4 AAP level IIIB and 1 AAP level IIIC) in the United States. This trial was registered at Clinicaltrials.gov (NCT01158391) before the first patient was enrolled. This study was approved by the Institutional Review Board at each participating site: Institutional Review Board of Santa Clara Valley Medical Center, IRB # 10–022; Montefiore-Albert Einstein College of Medicine of Yeshiva University IRB, IRB # 12-12-427; Baptist Health System Institutional Review Board, # BHS130010; Cook Children's Health Care System Institutional Review Board, # 2009–058. Written informed consent was obtained for this study.

### Participants

**Fig 1:**
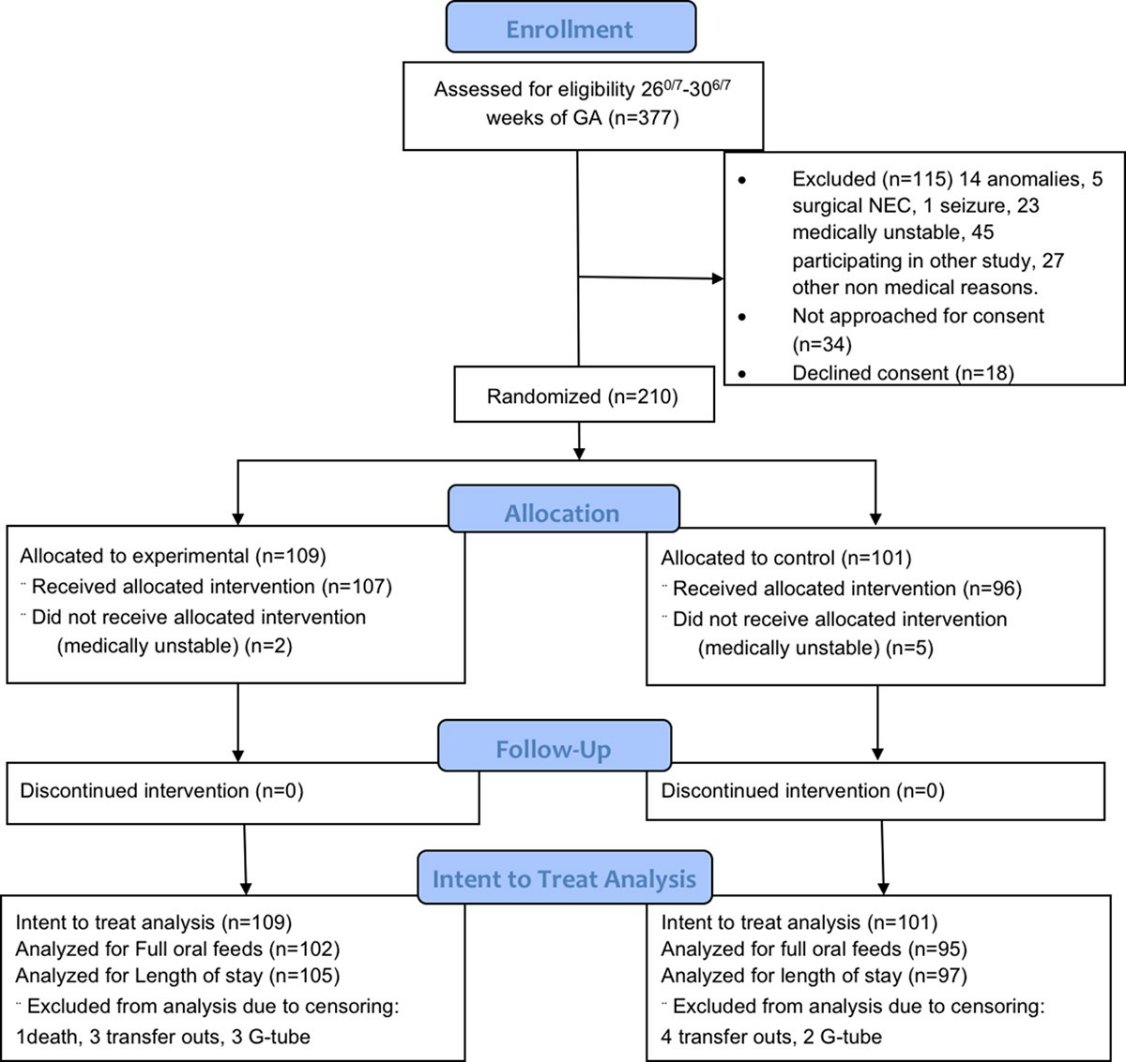
CONSORT flowchart—study subject eligibility and enrollment.

Preterm infants born at 26 0/7-30 6/7 weeks gestation were included (Fig 1). Infants born between 24 0/7–25 6/7 weeks gestation were not included as originally planned because they were medically too unstable to initiate study intervention prior to 32 weeks of gestational age (GA) as specified in the protocol. Infants with chromosomal or congenital anomalies, meningitis, seizures, necrotizing enterocolitis ≥ Bell stage 2, vocal cord paralysis, and those who were already taking feeds orally were excluded. Study investigators obtained written informed consents from parents of eligible infants and enrolled study patients at ≥ 28 0/7 weeks postmenstrual age (PMA). Infants were randomized to the experimental or control group using a centrally generated four block randomization log using SAS v. 9.1 statistical software, stratified at each center by GA subgroup of 26 0/7-28 6/7 weeks and 29 0/7-30 6/7 weeks. The central coordinator assigned the randomization number after a study patient was enrolled. Infants of multiple births were randomized to the same intervention group.

### Study interventions

**Fig 2:**
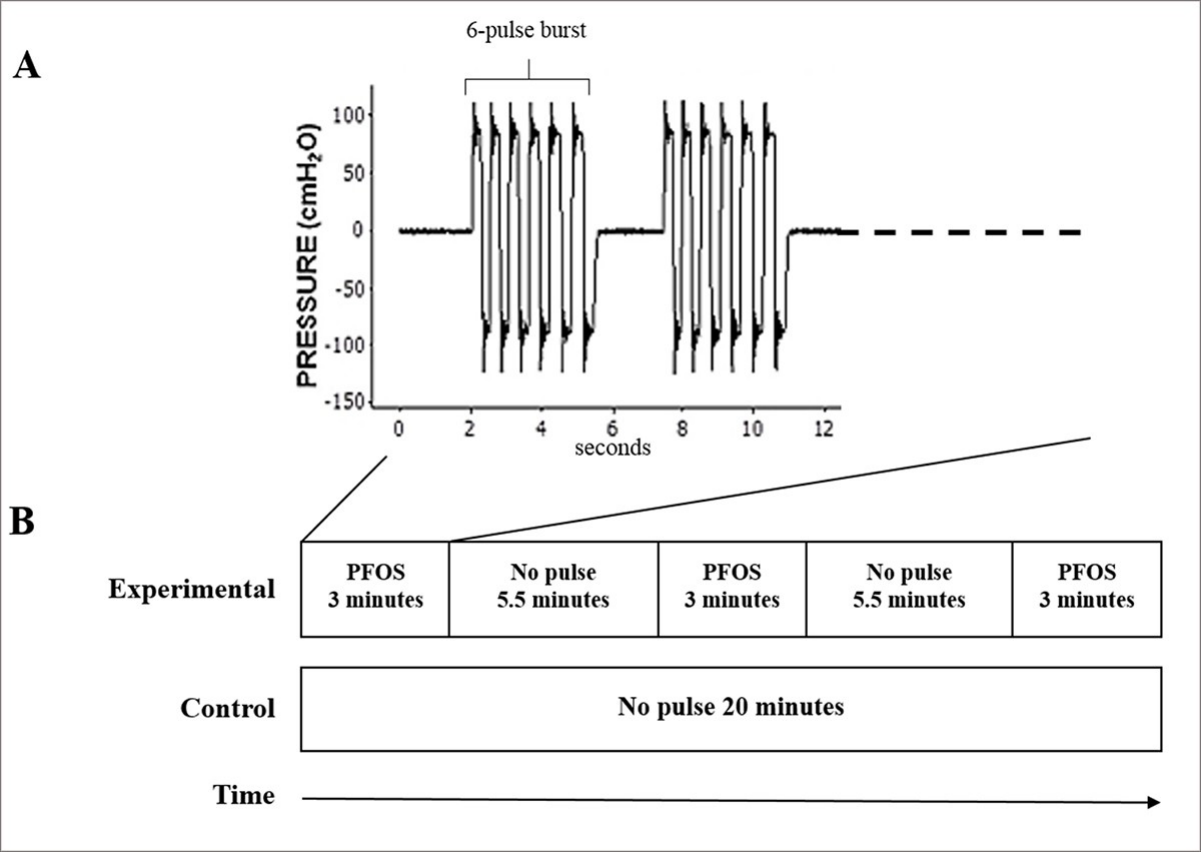
Intervention sessions. **(A)** The NTrainer pulses are patterned as 6-cycle bursts followed by 2-second pause periods. (**B)** An experimental session: three 3-minute PFOS epochs and two 5.5-minute non-stimulation epochs; a control session: a 20 minute no pulse period. PFOS: patterned and frequency-modulated oro-somatosensory stimulation.

We followed the previously published NTrainer oral training regimen [36]. Infants in the experimental group received the patterned frequency-modulated oro-somatosensory stimulation delivered via the NTrainer System (Innara Health Inc., Olathe, KS, USA) through a pacifier interface (Philips Avent Soothie Pacifier, Stamford, CT, USA). The pneumatic stimulator in the NTrainer system generates a series of pulses patterned as 6-cycle bursts followed by 2-second pause periods (Fig 2A), which transforms the pacifier into a pulsating nipple. Each NTrainer pulse was frequency modulated (0 to 16 Hz) through the dynamic intraluminal pressure changes to stimulate oral facial nerves [38]. A PFOS training session lasted 20 minutes and consisted of three 3-minute PFOS epochs and two 5.5-minute non-stimulation epochs during which the pneumatic stimulator was switched off (Fig 2B). Infants assigned to the control group received oral suck training using the same type of Soothie pacifier that was not pulsatile during the entire 20-minute session (Fig 2B). Infants were continuously monitored during study sessions, and interventions were halted if an infant showed signs of intolerance to the intervention or instability in vital signs. Both experimental and control interventions were performed by non-blinded occupational and physical therapists or a small group of trained clinical nurses. Other NICU staff including physicians, nurse practitioners and the non-study clinical nurses were blinded. The same NTrainer System was set up at bedside during both experimental and control interventions.

**Fig 3:**
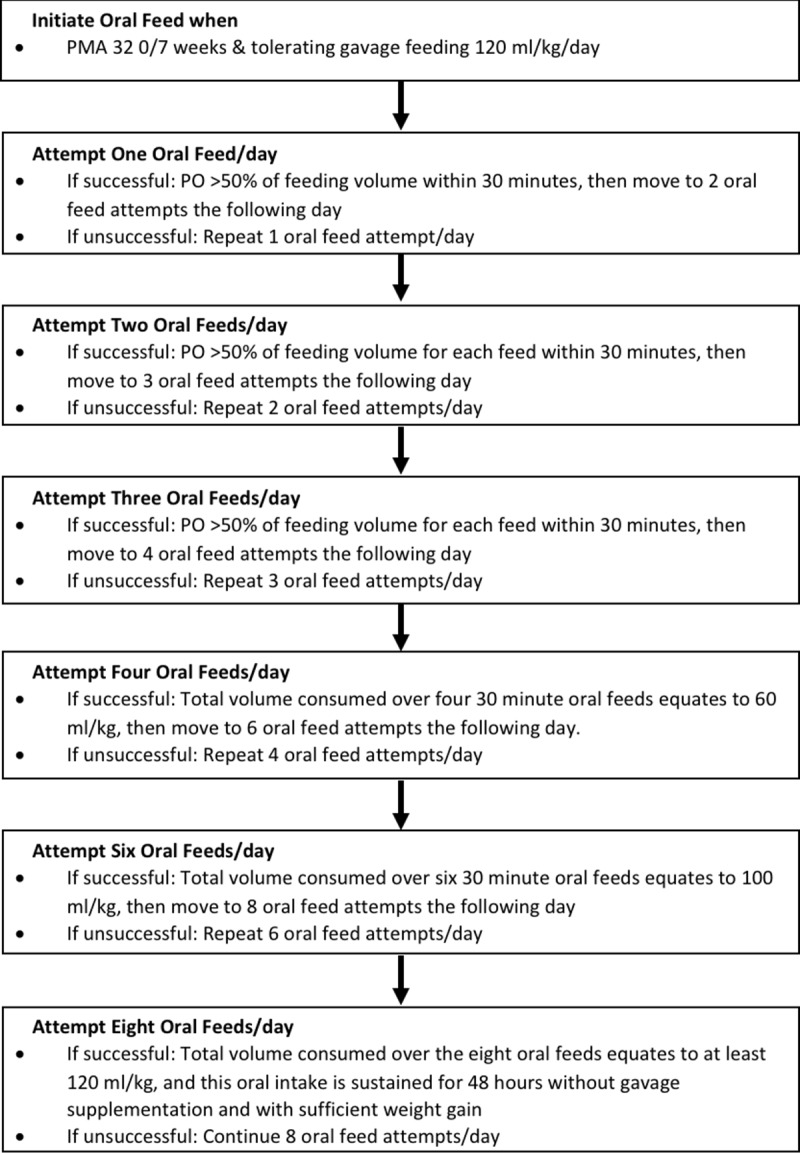
Oral feeding advance protocol.

Interventions began between 30–32 weeks postmenstrual age, once infants were tolerating enteral feeds and medically stable (i.e., not on any vasopressor, not mechanically ventilated, and FiO2 less than 40% if on continuous positive airway pressure or high flow nasal cannula ≥ 2 LPM). Infants received full volume gavage feeding during intervention sessions. When the infant’s mother was available for breastfeeding, intervention sessions were not performed. Interventions were performed up to 4 times daily to accommodate breastfeeding attempts. Interventions were stopped after a 2-week training period or when the infant reached full oral feeds, whichever came first. During interventions, if infants were inside isolettes they remained in the isolettes and were cradled in a supportive inclined position. Infants already weaned to open cribs received the interventions while being held in a feeding position on the lap of staff. Oral feeding was initiated after 31 6/7 weeks of gestation and advanced per oral feeding protocol (Fig 3), developed based on the previous publication [39]. If the infant was on high flow nasal cannula (≥ 2 LPM) or continuous positive airway pressure, oral feeding was initiated when FIO2 was <40%. Oral feedings were conducted following developmental care practice which included continuously assessing the infants’ autonomic and motor state, and responding in real time. Infants who showed feeding readiness, including alertness with adequate muscle tone, hand to mouth behavior, rooting or taking pacifier, were allowed to feed at their own pace for up to 30 minutes. If infants showed signs of fatigue or instability, feeding was stopped.

### Outcomes

The primary outcome of the study was the number of days from initiation of oral feeds to successful FOF by bottle or breastfeeding. Successful oral feeding was defined as no gavage supplementation and taking at least 120 ml/kg/day of milk (if bottle-fed) for 48 consecutive hours with sufficient weight gain. Secondary outcomes of the study included LOS in NICU, PMA at FOF, PMA at discharge, weight gain (g/kg/day) using the published exponential model [40], and head growth (cm/week) from birth to discharge. Infants were censored for the primary outcome if they died prior to achieving FOF, were discharged home with a gastrostomy tube, or were transferred to another hospital before reaching FOF. Infants were censored for the secondary outcome of LOS if they died or were transferred to another hospital. The study protocol specified collecting data to assess the ability to breastfeed prior to discharge, however, we were unable to collect this data due to lack of consistent documentation. Neonatal outcomes included mortality, necrotizing enterocolitis, late onset sepsis, aspiration pneumonia, and chronic lung disease.

### Sample size and statistical analysis

The sample size calculation used the time to FOF (Mean 23 and SD 12 days) which was reported by Simpson et al [39]. The sample size of 210 infants (105 in each group) was calculated to detect a 5-day difference in time to reach FOF between the experimental and control groups, with a two-sided alpha of 0.05 and 80% power (β = 0.2) with allowance for up to 10% censoring of the primary outcome. Subgroup analysis in the GA groups of 26 0/7-28 6/7 weeks and 29 0/7-30 6/7 weeks was planned *a priori*. Data were collected by investigators at each study site and audited by an independent agency (Cosgrove Consulting, Blue Springs, MO). Intent-to-treat analysis (n = 210) was performed to compare the experimental and control groups. Outcomes between experimental and control groups were compared using generalized estimating equations [41] (StataCorp 2013, StataCorp LP, TX, USA) with a linear link for continuous variables (time to FOF, LOS, PMA at FOF, PMA at discharge, weight gain and head growth), and a logistic link for binary variables (mortality and morbidities), with clustering of errors at the familial level to account for randomization of multiple births to the same intervention group. Time-to-event analyses (time to FOF, LOS) were conducted using Cox proportional hazards models, which also clustered errors at the familial level to account for the randomization of twins and triplets to the same intervention group. This clustering of errors is consistent with previous studies that randomize twins to the same study arm [42]. Time-to-event analysis (time to FOF, LOS) was illustrated as Kaplan-Meier curves.

## Results

### Subject enrollment

There were 262 eligible infants and 210 were randomized (experimental group n = 109; control group n = 101), including 38 multiple births (32 twins and 6 triplets) (Fig 1). Seven infants (2 experimental, 5 control) did not receive study interventions due to medical instability. There was 6.2% censoring for time to FOF; 7 in the experimental group (1 died, 3 discharged with gastrostomy tube feeding, 3 transferred) and 6 in the control group (2 discharged with gastrostomy tube feeding, 4 transferred). There was 3.8% censoring for LOS; 4 in the experimental group (1 died, 3 transferred) and 4 in the control group (transferred).

### Demographics

There was no difference in the baseline characteristics of the infants between experimental and control groups in birth weight, GA, sex, race, ethnicity, antenatal steroid exposure, or study parameters between the experimental and control groups (Table 1).

**Table 1:** Patient characteristics and study parameters.

	Experimental	Control	*P*
	*N* = 109	*N* = 101	
Gestational Age, weeks, Median (range)	28.9 (26, 30.9)	28.6 (26, 30.9)	0.2
Birth Weight, grams, Median (range)	1170 (483, 1980)	1140 (539, 2000)	0.4
Antenatal Steroids, %	82.6	86.1	0.5
Male, %	57.8	47.5	0.1
Ethnicity			1.0
*Hispanic*, %	53.2	54.5	
*Non-Hispanic*, %	40.4	39.6	
*Unknown*, %	6.4	5.9	
Race			0.8
*White*, *%*	53.2	51.5	
*Black*, *%*	24.8	19.8	
*Asian*, *%*	2.8	4	
*Other*, *%*	2.8	5.9	
*Unknown*, *%*	16.5	18.8	
PMA at initiation of oral feeds, weeks, Mean (*SD*)	32.6 (0.7)	32.7 (1.2)	0.4
**Study Parameters**	***n* = 107**	***n* = 96**	
PMA at initiation of study, weeks, Mean (*SD*)	30.7 (1.3)	30.6 (1.3)	0.6
Number of interventions per subject, Mean (*SD*)	32.7 (7.4)	33.8 (6.5)	0.3
% of interventions completed^a^, Mean (*SD*)	96.8 (4.9)	97.4 (4.6)	0.4
% of interventions on CPAP or NC^a^, Mean (*SD*)	65.4 (41.8)	58.4 (42.3)	0.2
% of feeding protocol deviations^a^, Mean (*SD*)	32.5 (18.4)	32.5 (19.7)	1.0

Abbreviations: PMA—postmenstrual age, *SD*—standard deviation, CPAP—continuous positive airway pressure, NC—nasal cannula.

^a^ % is calculated within each subject, and then group means of the individual %’s are computed.

**Fig 4:**
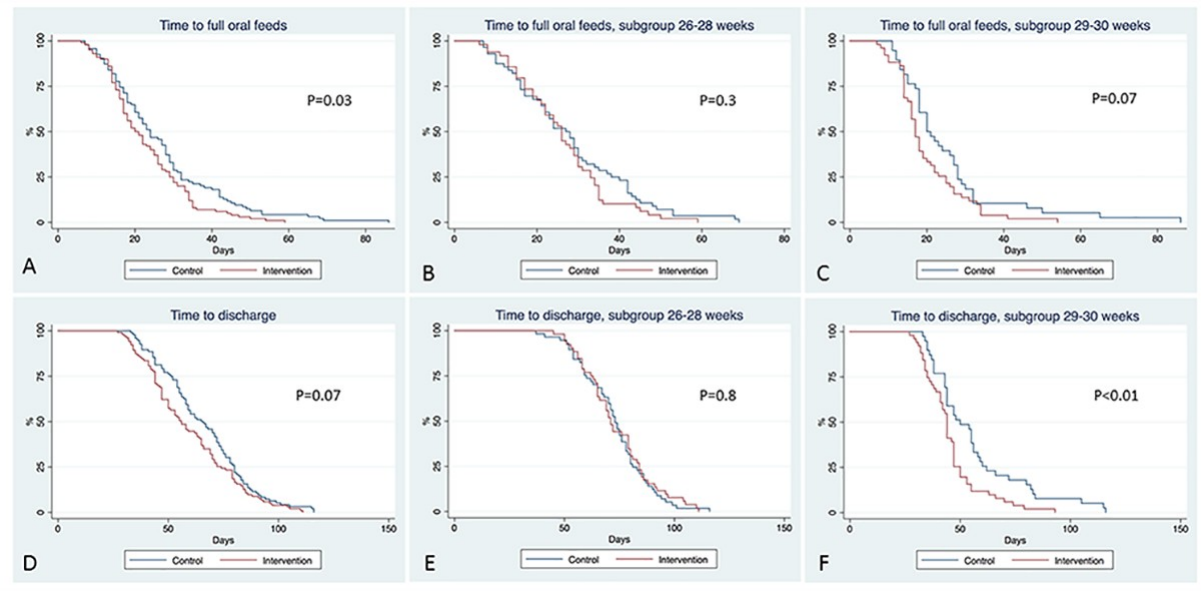
Kaplan-Meier curves for time to reach full oral feeds and length of stay.

Compared to the infants in the control group, infants in the experimental group showed significant reduction in time to FOF and LOS (Table 2). Time-to-event analysis showed that there was a significant reduction in time to FOF but not in LOS (Table 2, Fig 4). There is a suggestion of reduction in both PMA at FOF and PMA at discharge in the experimental group, although it did not reach statistical significance. In the 29–30 weeks GA subgroup (Table 2, Fig 4), infants in the experimental group had a significant reduction in LOS and PMA at discharge. This difference was not observed in the 26–28 weeks GA subgroup (Table 2).

**Table 2:** Study outcomes.

	Experimental Mean (*SD*)	Control Mean (*SD*)	Difference (95% CI)	*P*^a^	Hazard Ratio (95% CI)	*P*^b^
	*N* = 109	*N* = 101				
**Entire Cohort**				** **		** **
*Reached full oral feeds*	*n = 102*	*n = 95*		** **		** **
Time to full oral feeds, days	22.9 (10.5)	27.0 (14.8)	-4.1 (-7.8, -0.2)	**0.04**	1.37 (1.03, 1.82)	**0.03**
PMA at full oral feeds, weeks	35.8 (1.7)	36.4 (2.3)	-0.6 (-1.2, 0.01)	0.05		
*Discharged home from study NICU*	*n = 105*	*n = 97*		** **		** **
Length of stay, days	59.8 (20.2)	65.8 (19.8)	-6.0 (-11.9, -0.2)	**0.04**	1.30 (0.98, 1.72)	0.07
PMA at discharge, weeks	37.3 (2.1)	37.9 (2.4)	-0.6 (-1.3. 0)	0.05		
**GA Sub Group (26–28 weeks)**	***n* = 56**	***n* = 60**				
*Reached full oral feeds*	*n = 50*	*n = 56*		** **		** **
Time to full oral feeds, days	26.0 (11.2)	28.2 (14.8)	-2.2 (-7.5, 3.1)	0.4	1.24 (0.84, 1.83)	0.29
PMA at full oral feeds, weeks	36.1 (1.8)	36.6 (2.2)	-0.5 (-1.3, 0.4)	0.3		
*Discharged home from study NICU*	*n = 53*	*n = 57*		** **		** **
Length of stay, days	73.6 (15.9)	72.5 (15.6)	1.1 (-7.1. 5.0)	0.7	0.94 (0.64, 1.37)	0.75
PMA at discharge, weeks	38.0 (2.2)	38.0 (2.0)	-0.02 (0.8, -0.8)	1.0		
**GA Sub Group (29–30 weeks)**	***n* = 53**	***n* = 41**				
*Reached full oral feeds*	*n = 52*	*n = 39*		** **		** **
Time to full oral feeds, days	20.0 (9.0)	25.1 (14.9)	-5.1 (-10.4, 0.1)	0.06	1.49 (0.96, 2.29)	0.07
PMA at full oral feeds, weeks	35.6 (1.6)	36.2 (2.3)	-0.6 (-1.5, 0.2)	0.2		
*Discharged home from study NICU*	*n = 52*	*n = 40*		** **		** **
Length of stay, days	45.6 (13.2)	56.2 (21.3)	-10.2 (-18.0, -3.0)	**0.006**	1.87 (1.23, 2.84)	**<0.01**
PMA at discharge, weeks	36.6 (1.8)	37.8 (2.9)	-1.2 (-2.2, -0.2)	**0.02**		

Abbreviations: CI–confidence interval, *SD*–standard deviation, PMA–postmenstrual age.

^a^ P-values were adjusted for cluster randomization of multiples.

^b^ P-values were obtained from Cox proportional hazards models

The X axis is the time in days to reach the outcome and Y axis is the percentage of infants that reached the outcome. Time to reach full oral feeds in the whole study cohort (A), in the 26–28 weeks GA subgroup (B) and in the 29–30 weeks GA subgroup (C). The length of hospital stay in the whole study cohort (D), in the 26–28 weeks GA subgroup (E) and in the 29–30 weeks GA subgroup (F).

### Neonatal outcomes

There was no difference between experimental and control group neonatal mortality or morbidities (Table 3). One infant in the experimental group died of liver failure, unrelated to the intervention. The percentage of infants with chronic lung disease was 17% in the 26–28 weeks GA subgroup (9 in experimental and 10 in control, p = 1.0) and 3% in the 29–30 weeks GA subgroup (0 in experimental and 3 in control, p = 0.08).

**Table 3:** Neonatal outcomes.

	Experimental	Control	*P*^a^
	*N* = 109	*N* = 101	
Growth velocity, g/kg/day, Mean (*SD*)	12.6 (2.3)	12.9 (2.2)	0.3
Head growth, cm/week, Mean (*SD*)	0.71 (0.2)	0.71 (0.2)	1.0
Death, %	1	0	n/a
Aspiration pneumonia, %	0	0	n/a
Necrotizing enterocolitis, %	0	0	n/a
Late onset infection, %	4	6	0.4
Chronic lung disease, %	10	15	0.3

^a^ P-values were adjusted for cluster randomization of multiples.

## Discussion

This multicenter clinical trial showed that infants who received PFOS attained FOF four days faster than the control group and in the 29–30 weeks subgroup, those who received PFOS were discharged from NICU ten days earlier than the control group. This intervention was well tolerated and did not have an adverse impact on growth, neonatal mortality, or morbidity.

Infant sucking may be nutritive associated with swallowing milk or non-nutritive involving minimal swallowing except for infants’ own saliva [43–47]. Both types of suck involve the oral-pharyngeal neuromuscular system and are controlled by a neural network known as central pattern generators [3, 48]. Maturation of non-nutritive suck (NNS) has been shown to be a positive predictor for nutritive feeding performance [49]. A safe and effective oral feeding requires coordination between swallow and respiration [50–52]. Immaturity and associated respiration complications, such as respiratory distress syndrome and chronic lung disease, increase preterm infants’ risk for aspiration [53–55]. Therefore, non-nutritive oral interventions are commonly performed on preterm infants before they are able to orally feed safely. These interventions have been shown to have multiple beneficial effects on feeding development. They accelerate maturation of the central pattern generators [3], improve sucking skills and oral feeding performance [27–31]. Swallow practice with small milk boluses enhanced preterm infants oral feeding [32]. Although infants are not fed with milk orally during non-nutritive interventions, oral stimulation may increase saliva production and swallowing practice, which may facilitate synchrony between swallowing and breathing. A study conducted by Fucile et al. [56] has provided direct evidence that oral sensorimotor intervention facilitates suck-swallow-respiration coordination. Behavioral state is an important clinical parameter for assessing oral feeding readiness and performance [57,58]. Several studies have shown that sucking on pacifiers helped infants achieve and sustain a quiet alert state prior to and during oral feeding and improved their feeding readiness and efficiency [59–63]. Time to transition from gavage to FOF is a commonly used outcome in studies of infant feeding [4, 19, 26]. A 2016 meta-analysis showed that providing infants with a regular pacifier, compared with providing no intervention, significantly reduced time from initiation of oral feed to FOF (-2.2 days, n = 100) [3]. Importantly, more recent studies show that non-nutritive oral interventions shortened transition time from gavage to breastfeeding and increased breastfeeding rates [64–68]. Some of these effects were not observed consistently [32–35]. The discrepancies may result from differences in intervention methods, study protocols (i.e., timing, duration, and frequency of interventions) and patient populations, small sample sizes that are not powered to reach statistical significance, or operator-dependent variations [3, 19].

Previous studies have shown that PFOS, delivered by NTrainer through a pulsatile pacifier interface, is more effective than a non-pulsatile pacifier in facilitating NNS maturation in preterm infants [18, 19, 25]. The regular non-pulsatile pacifier training depends on preterm infants’ suck movements which are immature, low amplitude, low frequency (0–2 Hz), and of poor consistency. In contrast, the PFOS provides consistent oral-somatosensory stimuli which mimic the amplitude and frequency of mature NNS bursts [36]. The efficacy of PFOS also critically depends on the characteristics of its frequency modulation (0–16 Hz) [38], which targets the touch frequency spectrum of orofacial mechanoreceptors (0–100 Hz) [69, 70]. This broader stimulation spectrum may generate more effective sensory inputs in inducing neuroplastic changes in brainstem feeding centers and sensory and motor cortices [71. 72].

A large observational study showed that infants born at 26–30 weeks’ gestation, with routine care, attained FOF at 37–39 weeks PMA [73]. In our study, infants who received non-pulsatile pacifier NNS training reached FOF at 36 weeks PMA. Moreover, infants who received PFOS showed further reduction in time to reach FOF by 4 days. These infants were capable of taking sufficient oral feeds at a mean PMA of 35 weeks, a feeding competence comparable to infants born at 35 weeks’ gestation [73]. The previous studies of PFOS have mainly focused on the efficacy of PFOS on NNS development [36–38]. This multicenter RCT evaluated the effect of PFOS on nutritive feeding competency. Collectively, results from these studies have demonstrated a consistent positive effect of PFOS throughout oral feeding development, from establishment of NNS to more mature, nutritive feeding.

Our subgroup analysis showed minimum effects of PFOS on FOF and length of NICU stay in the 26–28 weeks GA subgroup (Table 2 and Fig 4). In contrast, a previous study has shown PFOS to be more effective in improving NNS and shortening of NICU stay in less mature infants (23–28 weeks) with chronic lung disease compared to more mature infants (30–34 weeks) with only respiratory distress syndrome or no respiratory complications [74]. Although both studies used the same PFOS entrainment regimen, the timing of initiating intervention was different: our study initiated intervention at 30–32 weeks PMA, while the previous study initiated intervention later, at 34–35 weeks PMA. Thus, the efficacy of PFOS may depend on the timing of intervention, targeting a critical period for feeding development. The timing of intervention in this study may be too early and or too short a duration for infants born at a younger gestation who are at a higher risk for delayed feeding development [53–55].

Independent oral feeding is an essential physiologic competency and often a limiting step for discharging preterm infants from the NICU [9]. A recent meta-analysis showed non-nutritive training with a regular pacifier, compared to providing no intervention, reduced LOS (-4.6 days, n = 501) [3]. In our study, in comparison to regular pacifier training, PFOS reduced LOS by 6 days. This reduction, however, did not reach statistical significance in the time-to-event, possibly due to the fact that our study was not powered to show this difference using Cox proportional hazards models. Future studies with larger sample size are needed to determine the effect of PFOS on LOS. In the 28–30 weeks subgroup, we observed a 10-day reduction in LOS. This suggests that the effects of PFOS may not be limited only to the oral system, but may also influence other developmental processes affecting LOS. Mature cardiorespiratory control is another essential physiologic competency for hospital discharge and apnea is a common reason for prolonging NICU stays [75]. Further studies are needed to determine whether PFOS affects maturation of respiratory control as well as other factors influencing infant and parental readiness for discharge.

This clinical trial was conducted in different hospital settings and enrolled infants of diverse racial ethnic backgrounds, making it generalizable in the study preterm infant population. In addition, clinical nurses, occupational and physical therapists performed PFOS interventions, demonstrating that it is feasible in clinical practice.

This study has several limitations. We were not able to study infants born before 26 weeks’ gestation because of their medical instability during the predefined time period of interventions. Apnea, bradycardia, and desaturation are common reasons for feeding intolerance and delayed NICU discharge, but we collected vitals only during training sessions to monitor safety of the interventions. Examining these data until time of discharge might allow evaluation of the impact of PFOS on cardiorespiratory stability. We did not collect data on breastfeeding, parental involvement, or parental readiness to bring their infant home. PFOS training produces effective oral sensory input and can be used as an adjunct tool for therapists and caregivers to support preterm infant oral feeding development. However, its clinical application should be in the context of providing individualized care that is appropriate for the infants’ overall physiological and behavioral development.

## Conclusions

PFOS supports feeding development and shortens time to full oral feeds in very preterm infants. It helps them achieve the normal milestone of independent oral feeding and shortens LOS. A better understanding of how GA, chronological age and lung disease interact with the genetic control of feeding development can further improve our ability to effectively utilize different feeding entrainments in clinical practice [76].

## Supporting information

S1 CONSORT Checklist (DOC)

S1 Study Protocol (PDF)
