# Supplementary material for: Patterned frequency-modulated oral stimulation in preterm infants: A multicenter randomized controlled trial
Source: PLoS One. 2019 Feb 28;14(2):e0212675. doi: 10.1371/journal.pone.0212675 (PMC6394921; doi:10.1371/journal.pone.0212675)
Supplement: S1 Study Protocol — (PDF) [file pone.0212675.s002.pdf]

Multicenter
Randomized Control Trial of Patterned Oral Somatosensory Entrainment Stimulation Program (NTrainer System®) for Shortening Time to Full Oral Feeding

Sponsored by:
Innara Health, Inc. (formerly KCBioMedix)
23733 West 83rd Terrace
Shawnee, KS. 66227

Clinical Study Sites			
**Site 1**<br><br>Santa Clara Valley Medical Center<br>751 South Bascom Avenue<br>San Jose, California 95128<br><br>**Principal Investigator**<br>**Dongli Song, M.D., PhD**<br>Associate Chief, Neonatology<br><br>dongli.song@hhs.sccgov.org<br>TL: Number: 408-885-5420<br>FX: Number: 408-885-5418<br><br>**Co-Investigators at SCVMC**<br>**Priya Jegatheesan**, M.D<br>Director, Well Baby Nursery<br><br>**Sunshine Weiss**, M.D.<br>Attending Neonatologist	**Site 2**<br><br>Cook Children's Medical Center<br>801 Seventh Avenue<br>Fort Worth, TX. 76104<br><br>**Principal Investigator**<br>**Jonathan Nedrelow, M.D.**<br>Associate Director Neonatology<br><br>jnedrelow@mac.com<br>TL: Number: 682-885-4283<br>FX: Number: 682-885-1656<br><br>**Co-Investigators at CCMC**<br>**Chanda Simpson, M.D.**<br>Associate Director Neonatology<br><br>**Megan McGuirk, M.D.**<br>Attending Neonatologist	**Site 3**<br><br>Montefiore Medical Center - Weiler<br>1825 Eastchester Road<br>Bronx, New York 10461<br><br>**Principal Investigator**<br>**Suhas M.Nafday, MD, MRCP, FAAP**<br>Director, Newborn Services<br><br>SNAFDAY@montefiore.org<br>TL: 718-904-4105<br>FX: 718-904-2659<br><br>**Co-Investigators at Weiler**	**Site 4**<br><br>Montefiore Medical Center - Wakefield<br>600 East 233rd Street,<br>Bronx, NY 10466<br><br>**Principal Investigator**<br>**Suhas M.Nafday, MD, MRCP, FAAP**<br>Director, Newborn Services<br><br>SNAFDAY@montefiore.org<br>TL: 718-904-4105<br>FX: 718-904-2659<br><br>**Co-Investigators at Wakefield**<br>**Sheri Nemerofsky, MD FAAP**<br>Director of Newborn Services<br>Wakefield Division<br>Assistant Professor of Pediatrics
Site 5<br>Duke Medical Center<br>Study Site Not Initiated	**Site 6**<br><br>North Central Baptist Hospital<br><br>San Antonio, Texas<br><br>**Principal Investigator**<br>Ahmad Kaashif, M.D	.	

SUMMARY

Purpose

To evaluate the effect of a patterned oral somatosensory entrainment stimulation program (NTrainer System®) on shortening the time to reach full oral feedings of preterm infants born between 26 and 32 weeks gestational age.

Summary of Problem

Prematurity represents a significant risk for abnormal brain development and is a public health priority in the United States. Over twelve percent of all live births occur prematurely (Buck P & Thiesen, 2006; Kuehn 2006). Extremely premature neonates commonly develop a severe nutritional deficit during the first weeks after birth (Clark et al., 2003). This early nutritional deficit affects weight gain, growth in length, and head growth. More importantly, poor growth has been associated with adverse long-term neurodevelopmental outcomes (Bloom et al., 2003; Ehrenkranz et al., 2006). One of the most significant obstacles to establishing good nutrition is the establishment of normal oral feedings. Oral feeding competency is one of the most frequent and challenging hurdles facing many premature babies (Mizuno & Ueda, 2006; Tosh, McGuire, 2006; Barlow et al, 2001).

Successful neuronal migration, assembly, and functional connectivity of the brain are central for the development of coordinated sucking, swallowing, and breathing, as well as for the development of speech, language, and other communication skills. Appropriate oral experiences are critical in the final weeks of gestation when the central patterning of suck and feeding skills are being refined. Interruption of these experiences may impair fragile syntheses of how the brain maps these functions. Often, premature infants have delayed or uncoordinated sucking, swallowing and breathing pattern, as well as poor state control restriction (Mizuno, Ueda, 2006; Tosh, McGuire, 2006; Barlow et al 2001; Pinelli & Symington, 2005; Cevasco & Grant, 2005; Lau & Kusnierczyk, 2001; White-Traut et al, 2005; Stumm et al., 2008). Resultant feeding problems lead to poor nutrition and growth, as well as developmental delay. These problems may persist well into early childhood and lead to significant delays in the emergence of speech-language production and abnormal neurodevelopment (Adams-Chapman, 2006; Adams-Chapman & Stoll, 2006).

The NTrainer System® is a newly developed FDA cleared device that provides patterned oral somatosensory stimulus to newborn infants. Previous studies have shown that the NTrainer System's® patterned oral somatosensory regimen facilitates the emergence of a stable non-nutritive (NNS) sucking patterns during a critical period of brain development of preterm infants in the extra uterine environment (Barlow SM et al., 2008, Poore et al., 2008).

The principle aim of this study is to evaluate whether the NTrainer System® patterned oral somatosensory entrainment enhances oral feeding maturation. NTrainer System® stimulation is hypothesized to yield multiple benefits to premature infants, including decreased transition time from gavage to full oral feeds, improved growth, and shortened length of hospital stays.

STUDY PROTOCOL

BACKGROUND

The development of sucking behaviors reflects neurobehavioral maturation and organization (Goldson1987). The early components of sucking have been demonstrated to occur in fetal life from about eight weeks post-menstrual age. Oral and gag reflexes first appear at about 12 weeks, and sucking appears at 24 weeks. Sucking and swallowing are present by 28 weeks; however, they are not coordinated until about 32 to 34 weeks. The spatiotemporal stability of an infant's non-nutritive suck (NNS) reflects the emergent rhythmic structure of the developing central pattern generators (CPGs) localized to ponytine and medullary centers in the infant's brainstem (Barlow & Estep 2006; Barlow 2009).

Appropriate oral experiences are critical in the final weeks of gestation when the central patterning of suck and feeding skills are being refined. Interruption of these experiences due to prematurity or medical interventions (i.e., orofacial trussing due to oxygen-ventilation therapy and feeding tubes) may impair fragile brain mapping of these critical functions (Estep et al. 2008; Stumm et al. 2008; Poore et al.2008).

From a clinical perspective, the ability to feed depends upon a coordinated suck, swallow, and breathing pattern. Often, premature infants have delayed or uncoordinated pattern of suck-swallow-breath and poor state control (Mizuno & Ueda, 2006; Tosh, McGuire, 2006; Barlow et al., 2001; Pinelli & Symington, 2005; Cevasco & Grant, 2005; Lau & Kusnierczyk, 2001; White-Traut, et al., 2005; Stumm et al., 2008). These conditions significantly affect their abilities to sustain full oral feedings. In the interim, infants are fed by gavage tube until they are mature enough to take milk directly from the breast or bottle.

NNS via a pacifier during gavage feeding facilitates the transition from gavage to breast and bottle feeding because NNS facilitates development of sucking behavior, allowing infants to achieve coordinated oral feeding faster (Pinelli & Symington, 2005; Rocha et al., 2007).

The NTrainer System®

In the past, all the NNS studies were conducted using a regular pacifier. Dr. Barlow's research group at the University of Kansas developed the NTrainer System®. This device provides two functions – 1) NeoSuckRT® Assessment and 2) NTrainerRT® patterned orocutaneous stimulation entrainment therapy.

NeoSuckRT® Assessment

Specialized data acquisition and digital signal processing software known as NeoSuckRT® allows ororhythmic activity assessment through a set of parameters: a) STI –STI NNS Index, b) Bursts/Minute c) Events/Burst d) NNS Events/Minute e) % NNN Events f) Total Events/Minute which quantifies spatial and temporal variability across kinematic trajectories (Poore et al., 2008).

NTrainerRT® Therapy

The patterned orocutaneous stimulus has been designed to mimic the temporal aspects of ororhythmic activity associated with the NNS, incorporating frequency modulation across the six-suck cycles. As shown in the plot that follows (figure 2), the synthetic NNS train is based on the results of modeling of the initial six-cycles of a NNS burst (compiled from modeling studies of N=1756 bursts among healthy preterm infants). This pattern is generated by a digitally controlled linear servo motor and pressurized actuator, which is pneumatically coupled by way of Luer tubing to the infant's preferred *Soothie®* silicone pacifier (Figure1-a)(Figure 1-b). The synthetic orocutaneous stimulus includes frequency modulation with the following sequence of cycle periods within the burst: 510, 526, 551, 580, and 626 ms producing 275 micron deflections of the Soothie™ nipple cylinder with 2-second interburst *pause periods*. This configuration transforms the pacifier into *pulsating nipple*, which resembles the spatiotemporal pattern of the NNS.

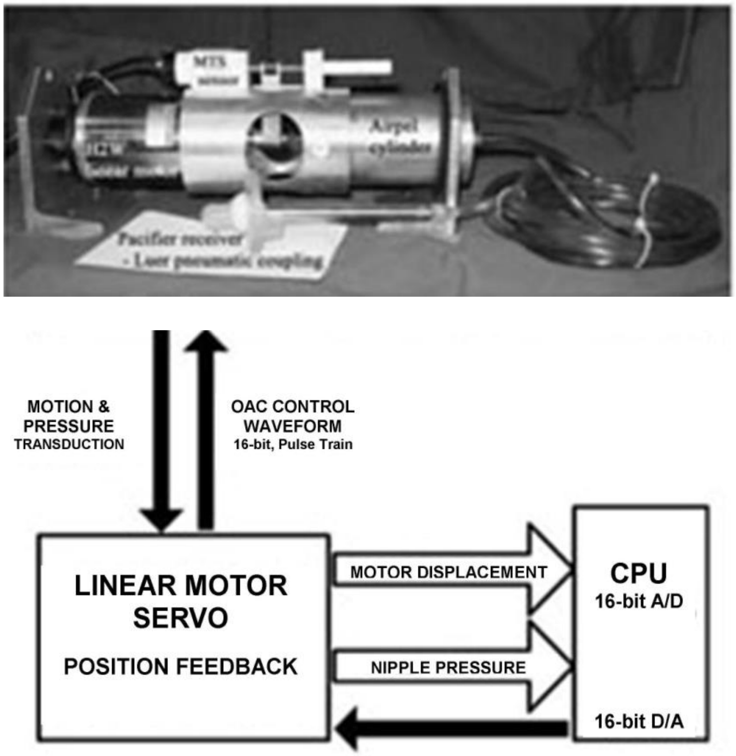

Figure 1-a. NTrainer System®.

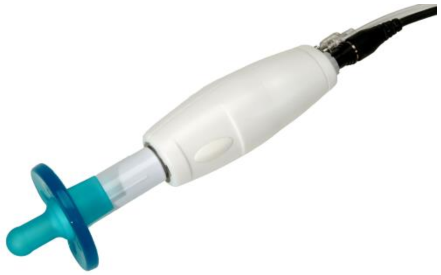

Figure 1-b. NTrainer System® hand piece with a silicone Soothie® pacifier.

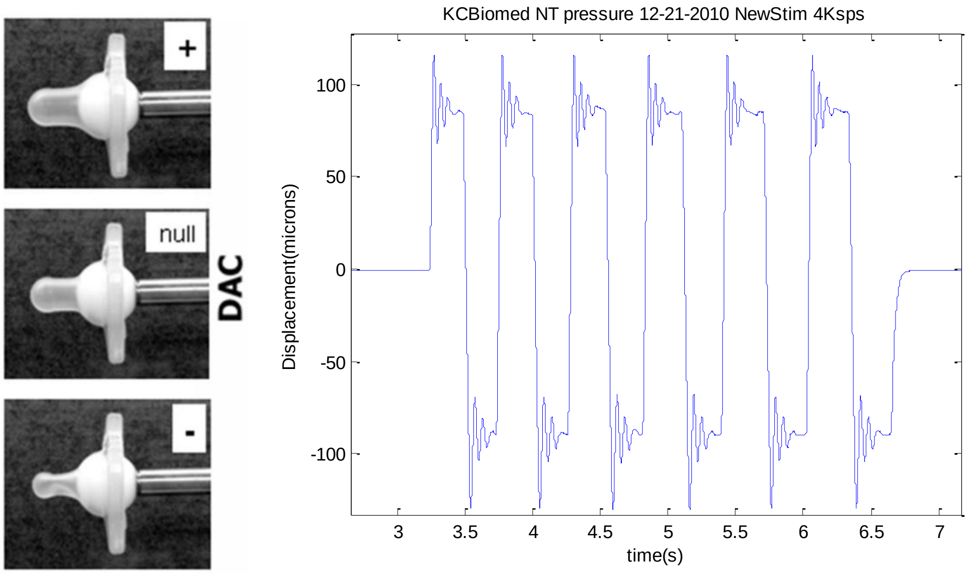

Figure 2. Positive and negative pressure appearance of a *Soothie*® silicone pacifier (exaggerated for visual appreciation), and a non-nutritive suck train pattern in time.

Recent studies in preterm infants have demonstrated that NTrainer System® entrainment enhances maturation of NNS both in healthy preterm infants and in preterm infants with respiratory distress syndrome (RDS) (Barlow et al., 2008; Poore et al., 2008). The preterm infants who have received this patterned orocutaneous stimulation outperformed their control groups on quantitative measures of NNS behaviors. Furthermore, the trained infants had an accelerated transition-to-oral feeding, surpassing their control counterparts; however, the trial was not powered to study transition-to-oral feed as the primary outcome.

This study is a multicenter randomized clinical trial to evaluate the effect of NTrainer System® entrainment on transition from gavage to full oral feeds during a critical period of brain development in infants born between 26 and 32 weeks gestational age. NTrainer System® stimulation is hypothesized to facilitate oral feeding maturation, improve growth, and shorten hospital stay.

HYPOTHESIS

The **primary aim** of this study is to test the hypotheses:

The *primary hypothesis* is that the preterm infants (26 to 30 weeks gestational age) who undergo the NTrainer System® training will transition to full oral feeds faster than the control group (i.e. the study group will be superior to the control).

The *secondary hypothesis* is that the infants in the NTrainer System® experimental group will have improved NNS parameters after completion of NTrainer System® training, will transition the first 100% oral feed, 100% oral feeds 4 times per day and improved growth at the time of full oral feeds and at the time of discharge.

The **secondary aim** of this study is to collect data on infants born after 31-32 weeks gestation who undergo the NTrainer System® training as pilot study to determine the effect size for future studies involving preterm infants.

STUDY POPULATION

Study subjects

After obtaining written informed consent from the parents and a consultation with the attending physician and nurses, preterm infants born between 26 and 32 weeks gestation will be enrolled in this study from the neonatal intensive care unit.

Inclusion Criteria

1. Gestational age 26-32 weeks at birth, as determined by Dubowitz/Ballad examination and/or obstetric ultrasound done at less than 15 weeks.
2. At least 28 weeks post menstrual age at the time of enrollment.

Exclusion Criteria

1. Chromosomal abnormalities, multiple congenital anomalies or any major congenital anomalies including craniofacial malformation, cyanotic congenital heart disease, gastroschisis, omphalocele, diaphragmatic hernia or other major gastrointestinal anomalies, major neurological anomaly.
2. Infants with history of surgical necrotizing enterocolitis (stage III).
3. Infants with vocal cord paralysis.
4. Infants with neonatal seizures.
5. Infants with meningitis at time of enrollment.
6. Infants who are nippling all feeds at the time of enrollment.

Randomization

A stratified blocked randomization technique will be used. The subjects will be randomized to the **Experimental Group** or the **Control Group and** stratified into three groups based on their GA:

26-28 and 6/7 weeks gestation,
29-30 and 6/7 weeks gestation,
31-32 and 6/7 weeks gestation. (Pilot Study – Discontinued Spring 2013)

A blocked randomization technique will be used to ensure approximately equal number of subjects are in each group. The randomization will be generated by SAS v. 9.1 statistical software program. Just prior to randomization, the inclusion and exclusion criteria will be reviewed for each candidate.

NTRAINER SYSTEM® INTERVENTION

Study Time line:

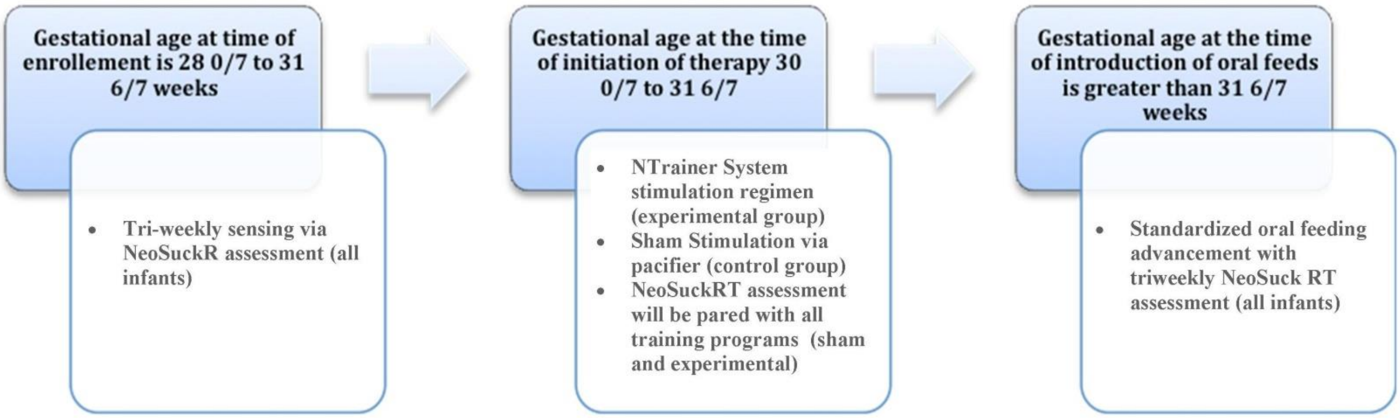

Infant Preparation-Positioning (A*pplicable to all assessment and stimulation/recording sessions)*
The infant will be cradled in a supportive inclined posture, swaddled, with limbs positioned at midline, and background/overhead lighting dimmed in the isolette area to promote eye contact with the tester. Infants may also be positioned for assessment and training while still remaining in their isolette as would be appropriate for their gestational age. The infant will remain connected to the usual NICU monitors at all times for observation of respiration, heartbeat and oxygen saturation by the nurse.

Figure 3. Preterm infant receiving NTrainer System® patterned orocutaneous stimulation

NeoSuckRT® Assessment
The development of nipple compression pressure dynamics associated with the NNS will be monitored using the NeoSuckRT® program. This assessment of NNS will occur 15 minutes prior to a gavage or oral feed that is not assigned to an NTrainer System® intervention. A 3-minute digital record of NNS activity will be sampled and the digitized record of NNS behavior will be subjected to quantitative indexing of NNS amplitudes and cycle periods within burst, using a specially designed NNS waveform discrimination algorithm. After the NeoSuckRT® procedure is completed, the infant will be fed by the attending nurse. If the baby is able to feed by mouth, the investigator may observe the feeding. Speech pathology, physical/occupational therapists, research coordinators, or staff nursing may perform the assessments.

All infants enrolled in the study (**experimental group** and **control group)** will first start on the NeoSuckRT® program**.** They will have at least two assessments including clinical assessment by the nursing staff or research staff and assessment using the NeoSuckRT® program prior to the study intervention. The NeoSuckRT® assessment will be done at least three times a week before, during and after the study intervention is completed until the infant reaches the primary outcome.

NTrainerRT® Therapy/Intervention

The interventions will be initiated simultaneously with tube (gavage) feeds as early as 30 weeks postmenstrual age. Before and during each training session the infant will be evaluated for the following criteria:

Criteria for Initiation of each NTrainer System® Therapy:

1. Not on continuous vasopressor medications
2. Feeding in previous 48 hours.
3. Not mechanically ventilated. If the infant is on CPAP or Nasal canula >2 liters per minute, then the FiO2 must be <40%.

Criteria for Exiting an NTrainer System® Therapy:

1. If during an intervention the baby experiences Apnea/Bradycardic/Desaturation events requiring nursing stimulation or intervention during the training--hold training for one training session.
2. If the trainings are required to be held for 3 consecutive sessions, hold training for 1 day (24hours).
3. If NTrainer System® interventions are held for 3 days (72hours) and if GA is <32 weeks when ready to restart therapy interventions, then complete another 10 days of therapy sessions.
4. If NTrainer System® interventions are held for 3 days (72hours) and if GA is ≥32 weeks when ready to restart therapy interventions, then complete the remaining NTrainer System® sessions for a total of 10 days of therapy sessions.
5. If NTrainer System® interventions are held for 3 days (72hours) and the infant is on full oral feeds when ready to restart therapy interventions then discontinue any remaining NTrainer System® therapy sessions.

The NTrainer System® patterned synthetic orocutaneous stimulation will be delivered through a NICU (*green*) Soothie® silicone pacifier. A Wee Soothie® (purple) may be used if the baby is under 32 weeks and demonstrating behavior indicating that the size of the NICU Soothie® is aversive to the baby.

Infants in the **experimental group** will receive the NTrainer System® patterned synthetic orocutaneous stimulation **three to four times daily** during the first 30 minutes of a tube (gavage) feeding session or 30 minutes before oral feeding for 10-days distributed over a two week period. A single NTrainer System® stimulation regimen consists of a series of epochs given by: 3 minutes NTrainer System® ON, 5.5 minutes NTrainer System® OFF, 3 minutes NTrainer System® ON, 5.5 minutes NTrainer System® OFF, and 3 minutes NTrainer System® ON.

Infants assigned to the **control group** will receive a sham stimulation program in which the infant will be offered the same type of NICU Soothie® pacifier coupled to the receiver tube and handpiece, but not to the NTrainer System®. Infants assigned to the **control group** will be offered the pacifier by gently placing the pacifier on the lips. The pacifier should not be forced into the mouth and should not be moved in any manner that would represent any form of patterned input such as moving the pacifier in and out, tapping on the lips or face or massaging the mouth or oral facial structure. The sham will be presented **three to four times daily** at the beginning of the first 30 minutes of a tube (gavage) feeding session or 30 minutes before oral feeding. For each session if the baby does not latch onto the pacifier when it is offered, the use of the sham pacifier for that session will be discontinued. However, the infant will remain cradled in a supportive inclined posture for the duration of session. The sham will be used for 10-days distributed over a two week period. All other preparatory, infant positioning and sampling conditions will be equal among groups. The healthcare team will continue to promote feeding and growth as the standard of care for babies in the control group.

Breast Feeding
Non-nutritive breastfeeding and nutritive breastfeeding practice will not be altered or changed by the NTrainer System® interventions. The availability of the mother will be taken into consideration for the scheduling of NTrainer System® interventions and no study intervention will interfere with breastfeeding behavior.

Safety
To help ensure the safety of the study participants, temperature, heart rate, oxygen saturation, and blood pressure will be recorded daily throughout the therapy regimen.

OUTCOME MEASURES

Primary Outcome Measure (Primary Study Endpoint) - Time to reach 100% full oral feedings:
Time (in days) from initiation of oral feedings to the first oral intake that results in 100% oral feeding (definition of 100% oral feeding: no gavage supplementation and taking at least 120 milliliters per kilogram per day for 48 consecutive hours of breast milk, fortified breast milk or preterm formula as ordered by the child's physician. Decisions regarding oral feeding advancement will be made using the oral feeding advancement protocol (Appendix B) developed based on the study done by Simpson at al (2002).

Secondary Outcome Measures
1. Feeding parameters:
    a. The time (days) from the initiation of the NTrainer System® training to the first successful and four successful oral feeds in 24 hours.
    b. Rate of milk transfer (ml/minute) at the time of full oral feeds—no gavage feeding.
2. Development of NNS parameters assessed by the NeoSuckRT® program
    a. STI – STI NNS Index
    b. Bursts/Minute
    c. Events/Burst
    d. NNS Events/Minute
    e. % NNN Events
    f. Total Events/Minute
3. Growth velocity from the initiation of NTrainer System® training to discharge.
    a. Weight Gain (grams per kilogram per day)
    b. Head Growth (centimeters per week). Occipitofrontal circumference - place measuring tape around the front of the head, above the brow and the occipital area. The measuring tape should be above the ears.
4. Length of hospital stay (days).
5. Necrotizing enterocolitis, aspiration pneumonia, and death events.
6. Ability to breast feed prior to discharge based on LATCH score.

During the study, adverse events will be monitored to ensure that the rate of reported events does not exceed the expected rate. Also, concomitant medications, concurrent procedures, therapies and protocol deviations will be documented.

STATISTICAL ANALYSIS

Sample Size Calculation
The principal study subjects are infants born between 26 and 30 weeks gestation. The sample size was calculated based on the alternative hypothesis that the preterm infants born before 31 weeks gestation in the experimental group will transition initiation of oral feeds to attaining full oral feeds 5 days earlier than

the control group. Data from Simpson et al, (2002) demonstrate that infants who underwent a standard protocol for early oral feeding advance transitioned to the full oral feeds at 23 ± 12 (Mean ± SD) days. All subjects will be followed until they attain full oral feeds. Therefore it is expected that the time to full oral feeds will be available for all or nearly all subjects (i.e. there will be little or no censoring of event times). Since the time to full oral feeds may be right skewed, the Wilcoxon Rank Sum test will be used as the primary analysis. We consider 5 days to be a clinically significant difference between the control and the experimental group. Thus, 105 infants in each group of a two-sided alpha of 0.05 yield 80% power (β=0.2) to detect a 5 day difference in the time to reach full feeds.

The percent of subjects with censored times are those leaving the trial prior to attainment of full oral feeds. Censored data will be assessed in a fully blinded way when 75% of the target sample size has completed the protocol. If rate of censoring is higher than 10% at this time, the planned use of the Wilcoxon Rank sum test will no longer be considered appropriate, and a log-rank test will become the primary analysis. Since the log-rank test has lower power, the sample size will need to increase to 160 subjects per group to achieve 80% power to detect a 5-day difference in median time to full oral feeds.

The infants between 31 and 32 weeks of gestation may attain full oral feeds much sooner compared to the younger gestation; therefore the effect size may vary considerably in this group of preterm infants. We will include the 25 subjects per group born between 31 and 32 weeks gestation to obtain pilot data on their effect size in order to calculate the sample size for this group, but the primary analysis will not include these subjects.

Data Analysis

The primary outcome of the study is the time to attain full oral feeds. All subjects will be followed until they attain full oral feeds. Wilcoxon Rank Sum test will be used to test for a difference between the two groups. A secondary, supportive analysis will be performed using the log-rank test, with the understanding that this test will likely be underpowered. As mentioned in the sample size section, in the event that the rate of censoring is higher than the 10% of evaluable subjects expected, the Wilcoxon Rank Sum test will not be considered appropriate and the log-rank test will be elevated to the primary analysis method.

The null hypothesis is that there will be no difference in the median times to full oral feeds in the experimental group versus the control group, this will be tested using a 0.05 two-sided significance level, with the following hypotheses:

$H_0 : M_{NT} = M_C$
$H_a : M_{NT} \neq M_C$

Where M_{NT} and M_c refer to the population medians in the NTrainer System® group and the control group, respectively.

The time to event will be measured as the number of days from the initiation of oral feedings to full oral feeding. Kaplan-Meier survival curves will be produced to illustrate the time of attainment of full oral feeds in the two groups. As a supportive analysis, a Cox proportional hazards regression model will be used to test for a difference in the time to attain full oral feeds between the two groups, while controlling for age group (26-27, 27-28, 29-30 and 31-32 weeks post menstrual age) and the presence of co morbid conditions that interrupt the feeding schedule.

Exploratory subgroup analyses of the primary outcome in each of the different gestational age groups (26-27, 27-28, 29-30 and 31-32 weeks post menstrual age) will be performed to evaluate for any difference in

the effect of the NTraining® at different gestational ages, and within groups having and not having co morbid conditions that interrupt the feeding schedule.

The secondary outcome variables of time from the initiation of the NTrainer System® training to first and fourth successful oral feeds will analyzed in the same manner as the primary outcome variable.

The rate of milk transfer at the time of full oral feeds will be compared between groups using an independent t-test. We will also compare the NNS parameters after the completion of NTrainer System® and at the time of full oral feeds using ***t*** tests.

Weight gain (gms/kg/day) and Head Growth (cm/wk) will compared using independent t-tests, provided the data are reasonably normal. If the rates are highly skewed, the Wilcoxon rank sum test will be used.

Binary neonatal morbidity variables such as necrotizing enterocolitis, pneumonia and death will be compared between the two groups using the Cochran Mantel Haenszel (CMH) test, controlling for age group.

The length of hospital stay will be compared between groups using the Wilcoxon rank sum test, since this variable is typically right skewed.

The ability to breast feed prior to discharge (based on LATCH score) is a binary variable, and as such will be analyzed using the Cochran Mantel Haenszel (CMH) test, controlling for age group.

The number of apnea/bradycardia/desaturation free days prior to discharge, and the number of apnea/bradycardia/desaturation with feeds per day after reaching full oral feeds will be summarized descriptively. Statistical analysis of these variables may not be warranted since the decision to discharge may be closely linked to these values, and regardless of treatment group once they reach a certain level the discharge may occur.

MONITORING

Site Monitoring
The Principal Investigators will manage the study to include site recruitment, collection of essential documents, monitoring, and close out.

Data Monitoring and Education
The patient will be considered to have completed the study, after attaining full, independent oral feedings for two days or after death, discharge, or transfer to a hospital not participating in the study.

Data will be collected using a case report form (Appendix C). Additional study-related documents, study references and information upgrades regarding the project will be available at this site.

If subject is transferred to another hospital following completion of the NTrainer System® intervention, we will have the ability to obtain follow-up data on outcomes.

A representative from KCBioMedix will monitor the progress of the study. The monitor will communicate with each center from the beginning and throughout the study to provide education and weekly review concerning the protocol, electronic randomization, CRF, and data forms for the trial. Monitoring will include 100% review of the primary outcome and secondary outcomes. It will be necessary for the monitor to review those portions of the patients' medical records pertinent to the study.

The monitor will complete clinical monitoring in accordance with Good Clinical Practice Guidelines (GCP).

Site Audits and Monitoring
Facility audit, study initiation audit, midterm audit, and study close-out audits will be conducted during the course of the study by an independent third party.

Interim monitoring
A Data Safety Monitoring Board (DSMB) will be formed for this study which will be an independent advisory board that is comprised of at least one statistician and an expert in the field who are not directly involved in the study. The DSMB will meet once a year or after 50% of the study subjects complete the study whichever comes first. The adverse event reports will be sent to the DSMB as mentioned in the adverse event reporting section. A study report including the subject enrollment, outcomes, adverse events, and protocol violations will be prepared by the study investigators and sent to the DSMB prior to the meetings. The study may be stopped early if definite harm is demonstrated or modified if there is no possibility of answering the research question. The study will not be stopped early for evidence of benefit.

Safety Monitoring
Safety of the study will be monitored by the DSMB. All safety outcomes data will be collected on the case report form and will be reported as adverse events (AE) and serious adverse events (SAE) from each NICU. DSMB will review all the **adverse events** annually and **the** serious adverse events as and when they occur.

Expected Adverse Event:
The anticipated adverse events are any respiratory instability as evidence by Apnea/ Bradycardia or Desaturations during the study intervention NTrainer System® therapy session. This will be submitted to the DSMB annually.

Serious Adverse Events
Death and Stage 3 NEC/ surgical NEC will be considered serious adverse events. This will be reported to the institutional IRB and the study DSMB within 24 hours. The sponsor, KCBioMedix will also be notified as required in their reporting policy.

Unexpected adverse events
Any unexpected serious adverse events that may be life threatening will be reported to the DSMB and the institutional IRB within 10 days of the event occurring.

Treatment Withdrawal Guidelines
Parents may withdraw their infant from the study at any time. The investigator may withdraw any infant who becomes clinically unstable (e.g., sepsis, stage II or III necrotizing enterocolitis). The healthcare team will then continue the standard of care treatment. Data collection will continue until the infant is discharged from the hospital even after withdrawal to be able to capture any unexpected adverse event related to the study intervention.

ETHICAL CONDUCT OF THE STUDY

The study will be conducted in accordance with the principles that have their origins in the Declaration of Helsinki, as well as International Conference on Harmonization (ICH) Good Clinical Practices Guidelines (GCP) and applicable federal and state regulatory requirements.

Written informed consent will be obtained from parents of the subjects prior to enrollment. Translators will be used for non English speaking parents and consents will be provided in their primary language (English, Spanish, Vietnamese). The harm to the subjects will be minimized by having strict criteria to stop the study intervention when there is evidence of intolerance.

INSTITUTIONAL REVIEW BOARD (IRB)

The protocol, including the informed consent form, must be reviewed and approved by the Facility Name Institutional Review Board prior to the initiation of subject recruitment. The IRB will be notified of subsequent protocol amendments by the principal investigator(s). In addition, progress reports will be submitted to the IRB by the principal investigator(s) as indicated by IRB guidelines, but at least annually.

RECORDS RETENTION

U.S. Federal law requires that a copy of all records (e.g., informed consent documents, source documents, investigational new device (IND) safety reports, test article dispensing records, etc.) which support case report forms for this study must be retained in the files of the responsible principal investigator(s) for a minimum of two years. This would occur following notification by the principal investigator(s) that all investigations at Facility Name NICU are completed, terminated, or discontinued. If the files need to be stored longer than two years, the Investigator(s) will be notified of the time frame. If the investigator(s) retires, relocates, or for other reasons withdraws from the responsibility of keeping the study records, custody must be transferred to a person who will accept this responsibility.

HARDWARE/SOFTWARE

The NTrainer System® has received FDA clearance to market with the indication for use as "reinforcing NNS in newborns and infants born prematurely." The NTrainer System® is a dedicated real-time data acquisition and analysis system.

PUBLICATION POLICY

All information is to be considered confidential. Any scientific publication regarding this study will be reviewed and agreed by all the participating parties (ie. SCVMC, CCMC, DUMC and KCBioMedix).

APPENDICES

A. Reference List
B. Standardized Oral Feeding Protocol
C. Data Collection Form
D. Protocol Deviation Form

Appendix A

Adams-Chapman I, Stoll BJ. Neonatal infection and long-term neurodevelopmental outcome in the preterm infant. *Curr Opin Infect Dis*. 2006;19:290-297.

Als H. A manual for naturalistic observation of the newborn (preterm and full term infants). In Goldson E, editor. Nurturing the premature infant, Developmental Interventions in the Neonatal Intensive Care Nursery. 1995; New York: Oxford University Press p 77-85.

Barlow SM, Dusick A, Finan DS, Coltart S, Biswas A. Mechanically evoked perioral reflexes in premature and term human infants. *Brain Res*. 2001;899:251-254.

Barlow SM, Estep M. Central Pattern Generation and the Motor Infrastructure for Suck, Respiration, and Speech. *J Com Disorders*, 2006;39, 366-380.

Barlow SM, Finan DS, Chu S, Lee J. Patterns for the premature brain: Synthetic orocutaneous stimulation entrains preterm infants with feeding difficulties to suck. *J Perinatol*, 2008;28, 541-548.

Buck P, Thiesen K. Increased preterm births impact U.S. infant mortality. Lippincotts Case Manag. 2006; 11:228-230.

Clark RH, Thomas P, Peabody J. Extrauterine growth restriction remains a serious problem in prematurely born neonates. *Pediatrics*. 2003;111: 986-990.

Ehrenkranz RA, Dusick AM, Vohr BR et al. Growth in the neonatal intensive care unit influences neurodevelopmental and growth outcomes of extremely low birth weight infants. *Pediatrics*. 2006;117:1253-1261.

Estep M, Barlow SM, Vantipalli R, Lee J, Finan D. Non-nutritive Suck burst parametrics in preterm infants with RDS and oral feeding complications. *J. Neonatal Nursing*, 2008;14, 28-34.

Goldson E. (19 Nonnutritive sucking in the sick infant. J Perinatol. 1987;7:30-34.

Kuehn BM. Groups take aim at US preterm birth rate. *JAMA*. 2006;296:2907-2908.

Lau C, Kusnierczyk I. Quantitative evaluation of infant's nonnutritive and nutritive sucking. Dysphagia. 2001;16:58-67.

Mizuno K, Ueda A. Changes in sucking performance from nonnutritive sucking to nutritive sucking during breast- and bottle-feeding. *Pediatr Res*. 2006;59:728-731.

Pinelli J, Symington A. Non-nutritive sucking for promoting physiologic stability and nutrition in preterm infants. *Cochrane Database Syst Rev*. 2005;CD001071.

Poore M, Zimmerman E, Barlow SM, Wang J, Gu F. NTrainer therapy increases suck spatiotemporal stability in preterm infants. *Acta Paediatrica*, 2008;97, 920-927.

Poore M, Barlow SM, Wang J, Lee J. Respiratory Distress Syndrome history predicts suck spatiotemporal index in preterm infants. *J Neonatal Nursing*. 2009; In press.

Rocha DA, Moreira MEL, Pimenta HP, Ramos JRM and Lucena SL. A randomized study of efficacy of sensory-motor-oral stimulation and non-nutritive sucking in very low birthweight infant. *Early Human Development*. 2007;83:385-388.

Simpson C, Schanler RJ, Lau C. Early introduction of oral feeding in preterm infants. *Peiatrics*. 2002;110: 517-522.

Smith A, Zelaznik HN. Development of functional synergies for speech motor coordination in childhood and adolescence. *Dev Psychobiol.* 2004;45:22-33.

Stumm S, Barlow SM, Estep M, Lee J, Cannon S, Gagnon K, Carlson J, Finan D. The relation between respiratory distress syndrome and the fine structure of the non-nutritive suck in preterm infants. *J Neonatal Nursing*, 2008;14(1):9-16.

Tosh K, McGuire W. Ad libitum or demand/semi-demand feeding versus scheduled interval feeding for preterm infants. *Cochrane Database Syst Rev*. 2006;3:CD005255.

White-Traut RC, Berbaum ML, Lessen B, McFarlin B, Cardenas L. Feeding readiness in preterm infants: the relationship between preterm behavioral state and feeding readiness behaviors and efficiency during transition from gavage to oral feeding. *MCN Am J Matern Child Nurs.* 2005;30:52-59.

Zimmerman E, Barlow SM. Pacifier stiffness alters the dynamics of the suck central pattern generator. *J Neonatal Nursing,* 2008;14(3), 79-86.

Appendix B – Standardized Oral Feeding Protocol

Feeding Protocol— for the advancement of oral feeding for both experimental and control groups. This protocol is developed based on the study done by Simpson at al. (2002).

<u>CRITERIA FOR THE INITIATION OF ORAL FEEDS:</u>

1. Infant will have received an enteral tube feeding volume of at least 120ml/kg/day for at least 24 hours prior to initiation of oral feeding.
2. Infants on high flow nasal canula (>2LPM) or CPAP must be in < 40% Fi02.

<u>Flow Diagram for the advancement of oral feeds:</u>

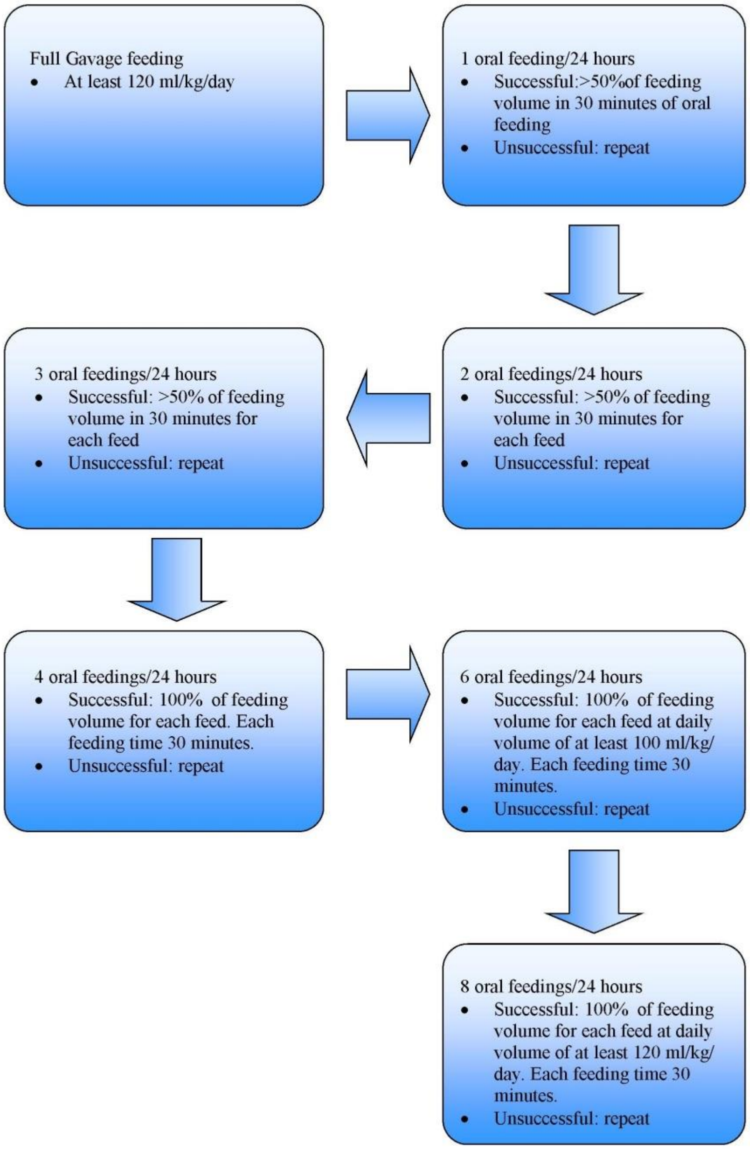

<u>Additional Features of a Successful Feeding</u> (all criteria must be met):

1. No oxygen desaturation event requiring the new use of or sustained increase (>10% Fi02) in baseline amount of supplemental oxygen.
2. No bradycardic events (heart rate less than 60 beats per min) requiring stimulation and/or multiple bradycardic (>3) events during a 30-minute feeding session (self resolving).
3. Breast feeding events will be considered successful if feeding time is >20 minutes.

If feeding was unsuccessful then the previous days number of oral feeding sessions are to be repeated until success is achieved. The nasogastric tube will remain in place during the oral feeding and any remaining volume of milk not fed in 30 minutes will be gavage fed.

Appendix C – Data Collection Forms (attached separately)

KCBioMedix, Inc. provides case report forms (CRFs) to study sites for use with each patient (control & experimental).

Appendix D – Protocol Deviation Form (attached separately)

Oral Feeding Trial
Protocol Deviation Report Form

Facility:____

Randomization Code:_______________________________

Printed on_____________Date_____________Time_____________

Question	Answers	Definition(s)
Date of Protocol Deviation	-	Date of Protocol Deviation
Time of Protocol Deviation	-	Time of Protocol Deviation
Type of Protocol Deviation	-Wrong Study Group<br>-Wrong Subject<br>-Other	Type of Protocol Deviation
If Held Parenteral Nutrition for Day, Indicate Dated	-	Type of Protocol Deviation – If Held Parenteral Nutrition for Day, Indicate Date
If Other, Indicate What Protocol Deviation is	-	Indicate other protocol deviation

Oral Feeding Trial

Physician Signature____Date______________
